# Deciphering Membrane Proteins Through Deep Learning Models by Revealing Their Locale Within the Cell

**DOI:** 10.3390/bioengineering11111150

**Published:** 2024-11-15

**Authors:** Mehwish Faiz, Saad Jawaid Khan, Fahad Azim, Nazia Ejaz, Fahad Shamim

**Affiliations:** 1 Department of Electrical Engineering, Faculty of Engineering, Science, Technology and Management, Ziauddin University, Karachi 74200, Pakistan; mehwish.faiz@zu.edu.pk (M.F.); fahad.azim@zu.edu.pk (F.A.); 2 Department of Biomedical Engineering, Faculty of Engineering, Science, Technology and Management, Ziauddin University, Karachi 74200, Pakistan; 3 Department of Biomedical Engineering, Balochistan University of Engineering and Technology, Khuzdar 89100, Pakistan; 4 Institute of Biomedical Engineering & Technology (IBET), Liaquat University of Medical and Health Sciences, Jamshoro 76060, Pakistan

**Keywords:** membrane protein, subcellular localization, cell, pseudo amino acid composition, deep learning models, proteomics

## Abstract

Membrane proteins constitute essential biomolecules attached to or integrated into cellular and organelle membranes, playing diverse roles in cellular processes. Their precise localization is crucial for understanding their functions. Existing protein subcellular localization predictors are predominantly trained on globular proteins; their performance diminishes for membrane proteins, explicitly via deep learning models. To address this challenge, the proposed study segregates membrane proteins into three distinct locations, including the plasma membrane, internal membrane, and membrane of the organelle, using deep learning algorithms including recurrent neural networks (RNN) and Long Short-Term Memory (LSTM). A redundancy-curtailed dataset of 3000 proteins from the MemLoci approach is selected for the investigation, along with incorporating pseudo amino acid composition (PseAAC). PseAAC is an exemplary technique for extracting protein information hidden in the amino acid sequences. After extensive testing, the results show that the accuracy for LSTM and RNN is 83.4% and 80.5%, respectively. The results show that the LSTM model outperforms the RNN and is most commonly employed in proteomics.

## 1. Introduction

A cell is a fundamental unit in the architectural design of living beings, which accommodates ample protein molecules. Most essential tasks for a cell’s survival are carried out by these proteins located in distinct locations [1]. The territory of the cell enfolds various organelles along with an outer membrane, which acts as a barrier between the extracellular and intracellular environments. The different organelles inside the cell are also enveloped by a definite membrane, and every organelle executes a unique task within the cell. The membrane proteins associated with each organelle have an exclusive function and, based on their specific locale, are categorized into plasma membrane proteins, internal membrane proteins, or organelle membrane proteins [2,3,4,5].

Membrane proteins (MPs) are an imperative part of every living cell that indulge in various metabolic pathways, and about one-third of all anticipated proteomes comprise membrane proteins. MPs also mediate the transport of solutes and the transduction of signals across membranes. Their main task is to anchor enzymes and other proteins to specific locations within the cell [6]. They also monitor inter- and intracellular activities by allowing the transportation of ions, metabolites, and larger molecules. They execute the propagation of chemical and electrical signals in pre-synaptic and post-synaptic neurons. The inclusion of lipids in the composition of MPs aids in maintaining the anatomical configuration of cells and cell organelles [7,8].

Specifying the locale of membrane proteins within the cell is pivotal in gaining insight into its biological processes and is a significant research area in molecular biology, bioinformatics, and proteomics. Locale Revelation of these proteins is challenging due to the hydrophobic nature of MPs [9], and very few machine learning-based predictor approaches are available for this task [10,11]. The identification of the residence of mem-brane proteins, particularly through deep learning (DL), is a new arena to be explored in bioengineering [12,13]. DL is notable in analyzing and evaluating intricate biological datasets and comprehending locale determination surpassing conventional techniques [2,14,15,16,17,18]. iDeepSubMito [16] and Deep Mito [17] employed deep learning algorithms to forecast the localization of sub-mitochondrial proteins. The suboptimal performance observed in [17] may be attributed to data insufficiency, impeding the adequate training of intricate architectures. Similarly, the efficacy of successive identification is constrained by the emphasis on biological features and the incorporation of evolutionary information [16]. DeepLoc [15] and DeepLoc 2.0 [18] are proposed to reveal the locale of proteins in eukaryotes, with DeepLoc 2.0 better outcomes. The drawbacks of [15] include the utilization of homology information as a predictive basis. Moreover, [18] encompass limitations associated with sequence length during training and potential biases stemming from dataset skewness. In 2021, Liao et al. used amino acid compositions and protein evolutionary matrices as feature extraction techniques to predict protein subcellular regions [19]. The method exhibits diminished performance on select datasets, notably dataset D4802, attributable to imbalances in sample composition and elevated correlations between subcellular locations within the training samples. All these techniques are based on DL and are either dedicated to proteins or mitochondrial proteins, pointing out the significance of the implication of revealing the locale of membrane proteins. These techniques encounter multiple issues, which are resolved by (a) selecting a dataset with less redundancy, (b) incorporating amino acid composition as the primary mode of extracting information, and (c) the sequence order of amino acids also considered for more precise indication. Moreover, empirical findings indicate that reliance solely on evolutionary information proves inadequate for protein classification into groups. The amino acid composition of proteins emerges as a pivotal factor in the accurate prediction of protein subcellular localization. One such approach is PseAAC, which accounts for both the sequence order and the amino acid content, which is particularly useful for modeling sequential information to enhance the predictive accuracy of localization models. Another feature engineering approach is the Gene Ontology Model. This method involves annotating proteins with a controlled vocabulary related to biological processes, cellular components, and molecular functions. GO annotations provide crucial contextual information about a protein’s function and localization, which is advantageous for localization studies. However, this approach is contingent upon the availability of numerous and accurate GO annotations for the proteins of interest. Similarly, physicochemical properties of amino acids focuses on the biophysical characteristics of amino acids (e.g., hydrophobicity, charge, and size). While it can provide insights into properties that influence membrane behavior, it lacks the capacity to capture sequence-specific details as effectively as PseAAC or GO models. Given these considerations, PseAAC may be the most effective approach due to its incorporation of both composition and order. Obtaining well-annotated data can be challenging, so GO models may not be suitable for every type of data. Physicochemical properties may serve as supplementary information but are unlikely to be effective as standalone predictors. Thus, PseAAC, without merging with any other characteristic, has consistently demonstrated superior performance compared to other methods [13,20].

Table 1 depicts the details of these deep learning algorithms employed for protein localization within the cell or mitochondria.

Several deep learning models are implemented for predicting the locations of proteins; however, only two predictors [21,22] specifically deal with membrane proteins by CNN, suggesting that this area must be investigated. Moreover, ref. [21] refers to a sequence-based predictor to disclose whether the protein is membrane protein or not, with no description of the location [21]. Similarly, MemBrain, uses a convolutional neural network to detect complex membrane-bound proteins in cryo-electron tomograms. It requires few training lines and achieves excellent accuracy with only one label [22]. The studies also reveal that the Mem Loci approach is implemented for disclosing the locale of membrane proteins by SVM only [10]. Based on this literature review, DL needs to be implemented in membrane protein localization as it can handle massive datasets to uncover intricate patterns, allowing for more precise predictions of the locations of these proteins. The following are the accomplishments in this planned work.

1. DL has been applied to the membrane proteins database of the MemLoci Approach.
2. The indulgence of pseudo amino acid composition in the auto-retrieval feature selection of DL has been completed for MP in the study. This feature extraction technique constructs the hydrophobicity and hydrophilicity of MP, thus granting explicit local information.
3. A comparison of RNN and LSTM has been proposed for membrane proteins excerpted from amino acid sequences, which is also a peculiar aspect of this research.

The findings in this study are divided into sections, each focusing on a particular analysis component. Section 2 demonstrates the methodology adopted with the details of the dataset, features extracted, and the RNN and LSTM models. In contrast, Section 3 illustrates the study’s results, and Section 4 states the conclusions drawn from the experiment.

## 2. Materials and Methods

This study aims to develop a compelling DL-based inter-classifier for local revelation of MP, and therefore an appropriate database with curtailed redundancy was chosen. The protein sequences were converted into a numerical representation through a feature extraction technique. To assess the model’s performance, the dataset was then split into training, validation, and testing sets accommodating 70%, 10%, and 20% of the data values, respectively. Deep learning architectures were then employed to investigate their efficacy in predicting the locale through amino acid sequences.

### 2.1. Dataset

The MemLoci approach made their benchmark dataset of membrane proteins available at https://mu2py.biocomp.unibo.it/memloci/ (accessed on 5 January 2023) and was curated by the Biocomputing Group at the University of Bologna from the SwissProt database with a threshold cut-off of 20%. The information of eukaryotic membrane proteins was extracted from from the SwissProt database, specifically selecting those with confirmed subcellular localization. Sequences annotated as ‘PROBABLE’, ‘POSSIBLE’, ‘BY SIMILARITY’, and ‘FRAGMENT’ were excluded to guarantee the reliability of the data. To minimize redundancy, proteins were categorized into similarity sets through all-against-all alignments utilizing BLAST, leading to the construction of a graph that connected sequence pairs sharing over 80% identical residues. The connected components of this graph were identified via a transitive closure algorithm, yielding 10,634 non-overlapping subsets, with only one representative protein retained from each subset. The resulting dataset of 10,634 sequences was further clustered based on low homology, specifically targeting proteins that aligned with an E-value ≤ 10^−3^. This process generated 1547 clusters, ensuring that proteins from distinct clusters exhibited low similarity, aligning with an E-value > 10^−3^ [10]. For this study, a subset of 3000 protein sequences was taken from the database of MemLoci, assimilating 1000 sequences from each category of membrane proteins, namely internal, organelle, and plasma membrane. These were downloaded in FASTA format, which is a text-based layout to illustrate protein sequences. Bioinformatics extensively utilizes this configuration to exchange and store biological sequencing data. A FASTA file usually presents one or more sequences, with each denoted by a header line and a sequence block. The sequence data is in the sequence block after the header line, which contains the description of the protein.

### 2.2. Feature Extraction

**Figure 1:**
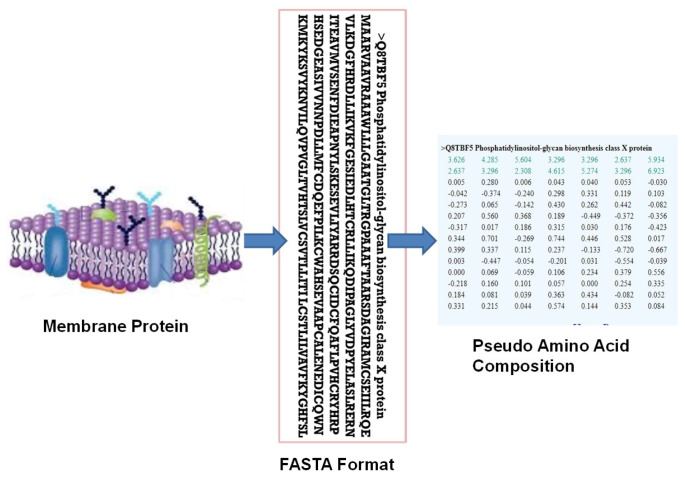
Transformation of membrane proteins sequences into pseudo amino acid composition.

During extracting features, the amino acid sequences obtained in FASTA format were transformed into pseudo amino acid composition (PseAAC). This feature was a set of numerical values representing the locations of amino acids in a protein sequence and their numerous physicochemical and structural characteristics. This transformation was performed using the Chou’s pseudo amino acid converter, which can be found at http://www.csbio.sjtu.edu.cn/bioinf/PseAAC/ (accessed on 4 April 2023). For this purpose, every protein FASTA format was entered in this converter to obtain the numeric feature value of the protein. This converter employed six characteristics, including hydrophobicity, hydrophilicity, pK1 (alpha COOH), pK2 (NH3), pI (isoelectric point), and mass of the side chain to represent properties of membrane proteins [25]. These characteristics had played a role in evaluating the influence of amino acid positions along the protein chain. The resulting PseAAC had produced a feature vector with more than 20 parameters. The initial 20 parameters corresponded to compositions of amino acids, while the subsequent ones encoded sequence order information and details about the amino acid location. These numeric values of PseAAC imitating the three distinct classes of membrane proteins accommodating internal, organelle, and plasma membranes were then input to the deep learning models. Figure 1 depicts the mechanism of transformation of the amino acid sequence into PseAAC.

### 2.3. Experiments and Evaluation

Python had been adopted to write code that uses the TensorFlow library on a Linux workstation. The pertinent libraries were initially imported for this study, including mat-plotlib.pyplot and seaborn for data visualization; numpy for numerical computations; os for interacting with the operating system; and pandas for data manipulation. Tensorflow was also used for deep learning, and plotly.express was imported for plotting. The data was then preprocessed, deleting several unnecessary columns and rows. Furthermore, the implementation incorporation of pseudo-amino acid codes was carried out along with some additional features, namely “mean”, “median”, and “standard deviation”. Data was labeled with three distinct protein classes containing 1000 proteins in each class.

Identified outliers were eliminated, and the dataset was then subjected to the computing algorithms of RNN and LSTM. These models were constructed using Keras, a high-level neural network API tailored for a classification task with three output classes.

#### 2.3.1. Recurrent Neural Network (RNN) Architecture

An artificial neural network with a specialized architecture called RNN architecture is built using feedback loops to handle sequential data efficiently. One element at a time, RNNs process input sequences, adjusting their internal state in response to the input at hand as well as the data saved from earlier stages. The provided equations demonstrate the output of the RNN model in forward propagation, where *Y_O_* is the final output.

(1) $$O_{1}=f\left(X_{11}*W_{R}\right)$$

(2) $$O_{2}=f\left(X_{12}*W_{R}+O_{1}*W_{1}\right)\;$$

(3) $$O_{3}=f\left(X_{13}*W_{R}+O_{2}*W_{1}\right)$$

(4) $$Y_{O}=f(O_{3})$$

#### 2.3.2. LSTM Architecture

Long Short-Term Memory (LSTM) is the deep learning framework particularly well-suited for the complex analysis of sequential data. This algorithm is widely used for sequence classification and time series prediction. These LSTM networks were created in response to the vanishing gradient issue, which makes it difficult for conventional RNNs to identify long-term dependencies in sequential data. Each unit or cell in an LSTM network has a memory component that helps it store information for extended periods of time. This feature helps the network recognize and remember pertinent patterns in sequential input. The input gate, forget gate, and output gate are the three gates that make up this memory mechanism. By selecting the data from the current input that should be stored in the memory, the input gate controls the amount of information that enters the cell. The forget gate allows the network to refresh the memory with new inputs or forget irrelevant information by controlling the retention or deletion of data from the cell’s memory. Lastly, the output gate controls the information flow from the memory of the cell to the output of the network or the subsequent time step. These following equations illustrate the operation of a single LSTM unit with the depiction of every gate and state involved.

(5) $$i_{t}=\sigma (x_{t}U_{i}+h_{t-1}W_{i})\;\;\;\;i_{t}=\mathrm{input}\;\mathrm{gate}$$

(6) $$f_{t}=\sigma (x_{t}U_{f}+h_{t-1}W_{f})\;\;\;\;f_{t}=\mathrm{forget}\;\mathrm{gate}$$

(7) $$o_{t}=\sigma \left(x_{t}U_{0}+h_{t-1}W_{0}\right)\;\;\;\;o_{t}=\mathrm{output}\;\mathrm{gate}$$

(8) $${\sim C}_{t}=tanh(x_{t}U_{g}+h_{t-1}W_{g})\;\;\;\;\sim C_{t}=\mathrm{candidate}\;\mathrm{for}\;\mathrm{cell}\;\mathrm{state}$$

(9) $$C_{t}=\sigma (f_{t}*C_{t-1}+i_{t}*{\sim C}_{t})\;\;\;\;C_{t}=\mathrm{updated}\;\mathrm{cell}\;\mathrm{state}$$

(10) $$h_{t}=\tanh\left(C_{t}\right)*o_{t}\;\;\;\;h_{t}=\mathrm{hidden}\;\mathrm{cell}\;\mathrm{state}$$

*x_t_* = input at current timestamp

*h_t_*_−1_ = output of the previous LSTM block

*W_x_* = weight for the respective gate(x) neuron

### 2.4. Layout of the Approach

The RNN algorithm was specifically designed to handle the complexities of sequential data to counteract the common problem of overfitting. It combined simple Recurrent neural network (RNN) layers with rigorous dropout regularization techniques. The three Simple RNN layers, each with a foundational unit count of 128 in the architectural framework, were initiated sequentially. The argument “return_sequences = True” was fundamental because it guaranteed the preservation of temporal information essential for thorough sequence comprehension. A deliberate digression ensued in the orchestration, presenting a dropout layer that came after the first RNN layer and had a regularization rate of 0.2. The overall goal of this augmentation was to improve the model’s capacity for generalization. This process continued with the addition of the second Simple RNN layer identified by an increased number of units (256) and the preservation of sequences. It also included a dropout layer set up with a regularization rate of 0.2. The third Simple RNN layer had a unit count of 256, marking a significant turning point. However, unlike its predecessors, this layer produced a single vector as output for every input sequence rather than returning sequences. To decipher intricate patterns buried in the data, another dense layer with 256 ReLU activations was introduced. In order to further improve the model’s representation, a second dropout layer with a 0.4 rate was purposefully added to this layer. The last architectural block included a dense layer with 64 units, which worked in concert with a dropout layer calibrated at a rate of 0.3. This complex symphony culminated in a three-unit output layer embellished with SoftMax activation as the final layer. Thus, leading to precise multi-class classification, demonstrating the model’s ability to translate intricate sequential patterns into understandable and significant results.

**Figure 2:**
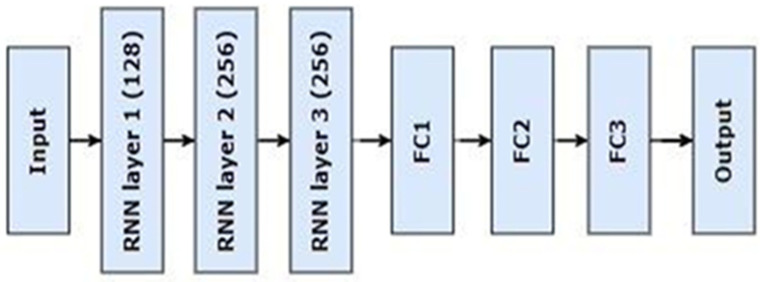
Compositional architecture of the proposed RNN model. FC1 = fully connected layer 1, FC2 = fully connected layer 2, FC3 = fully connected layer 3.

In Figure 2, the operation of the RNN network is illustrated using the general design of the algorithm. This model efficiently handled sequential data, and dropout layers enhanced its ability to generalize effectively. The SoftMax output ensured accurate predictions by accurately representing the class probabilities, effectively overcoming overfitting conditions.

**Figure 3:**
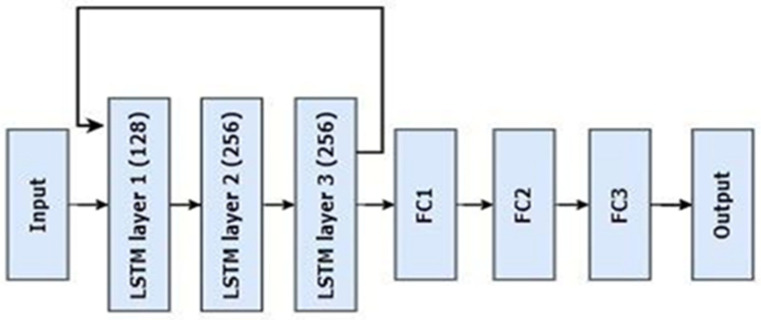
Compositional architecture of the proposed LSTM model. FC1 = fully connected layer 1, FC2 = fully connected layer 2, FC3 = fully connected layer 3.

The proposed model presented a 128-unit LSTM layer, an architectural design that enabled it to recognize and assimilate the complex patterns present in the sequential input data. In order to address the subtleties of univariate time series data, the input shape had been defined as (time_steps, 1). Moreover, the LSTM layer’s return_sequences = True setting made it capable of generating sequences in addition to individual outputs, which simplifies later tasks related to sequence processing. Dropout layers with rates of 0.2 and 0.3 were then chosen after the first and second LSTM layers, respectively, to strengthen the model’s robustness. The deliberate use of dropout layers accomplished two goals: improving generalization and serving as a strong barrier against overfitting. The effectiveness of the model was further enhanced by the deliberate use of several LSTM layers, each rigorously paired with dropout layers. The model was enabled to capture the complex temporal patterns and relationships typical of sequential data by use of this ensemble of LSTM and dropout layers. The architectural narrative expanded from the LSTM layers to a series of dense layers embellished with Rectified Linear Unit (ReLU) activation containing 256, 128, and 64 units in sequential order, as shown in Figure 3.

These dense layers were complemented by dropout regularization with rates of 0.4 and 0.3. The combination of dense and dropout layers enhanced the model’s ability to extract meaningful representations from the sequential input. The final dense layer employed a SoftMax activation function comprising three units, suggesting a multi-class classification task entailing three distinct output classes. This whole architectural design depicts that the LSTM model is a powerful tool for time series prediction and sequence classification tasks because of its designed layout and deliberate inclusion of dropout mechanisms. It is ready to decipher the complexities hidden inside sequential data. The layer-by-layer orchestration where each layer contributes differently to the overall performance of the model illustrates the complexities of modern deep learning frameworks in addressing difficult analytical problems. The specific details of all the layers and hyperparameters of the DL models are given in Table 2.

Table 2 indicates that all the Recurrent neural networks (RNNs), and Long Short-Term Memory networks (LSTMs) employed cross-entropy loss for multiclass classification. The loss function was consistent across these architectures, but its implementation varied based on the specific features of distinct networks. Mathematically, it can be expressed as:

(11) $$L=-\sum_{i=1}^{N}Y_{i}*log(y_{i})$$

*N* = number of classes, *Y_i_* = true class probability of class *i*, *y_i_* = predicted class probability of class *i.*

The ultimate aim of the model was to proficiently discern input membrane protein sequences and classify them accurately into either plasma membrane proteins, internal membrane proteins, or organelle membrane proteins. Each following model was compiled using sparse categorical cross-entropy as the loss function, Adam as the optimizer, and accuracy as the evaluation metric. To prevent overfitting and enable monitoring of the training process, the early stopping callback was set to monitor the validation loss, and tensor board callback was employed. If there was no improvement for five consecutive epochs, the model’s best weights will be restored. The training process involved feeding the model with X_train_reshaped and y_train data and validating on X_test_reshaped and y_test for 50 epochs with a batch size of 64. This configuration sought to create a robust model halting early if no progress was made and optimizing the classification task. The performance of each model was consistently gauged by comparing the training and validation datasets. Assessment for the model on its training data X_train_reshaped with y_train) while validation loss and accuracy were gauged via independent validation datum X_test_reshaped with y_{test}. Evaluation unveiled the efficacy of the model during the training phase by disclosing the training loss subsequent to the model’s training on a MemLoci approach dataset. Training accuracy, validation loss, and validity conformity represented these results in varying degrees that were always consistent within a given dataset or cross-dataset evaluation.

**Figure 4:**
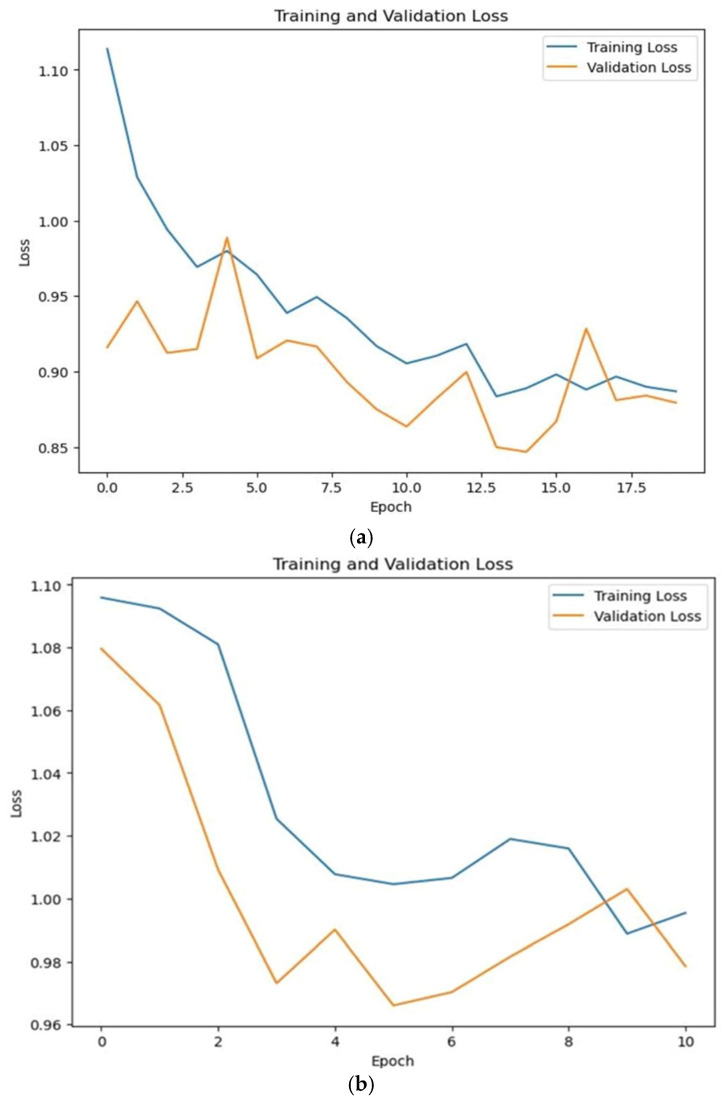
Loss evaluation of (**a**) RNN and (**b**) LSTM models.

Two different plots were drawn to showcase the progress of the training and validation processes, with the first graph charts in Figure 4 pinpointing the changes in the train loss values over an epoch. The *x*-axis denotes the progression of epochs, displaying numerical increments, whereas the *y*-axis represents the incurred loss. A thorough examination of this plot enables discernment regarding the appropriateness of the model’s learning process and the potential manifestation of underfitting or overfitting.

**Figure 5:**
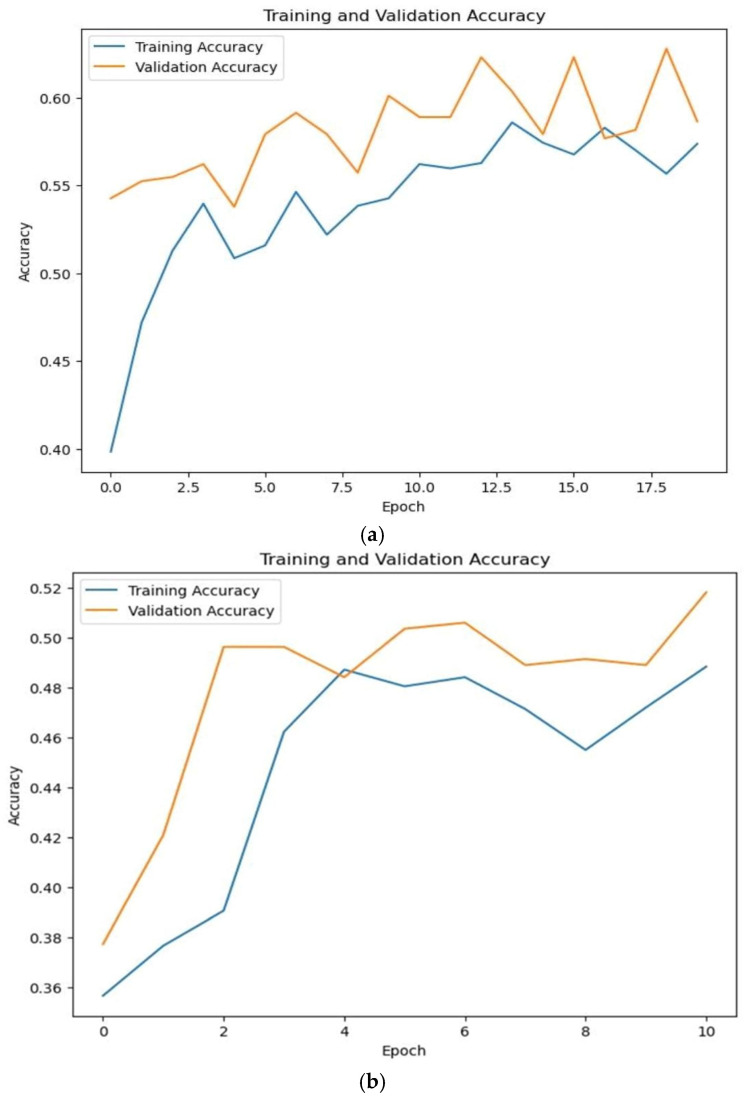
Accuracy evaluation of (**a**) RNN and (**b**) LSTM models.

A performance evaluation of the model during training is shown in Figure 5, showing each set’s accuracy trends as the number of epochs increased along the *x*-axis. Through scrutinizing the convergence of values on the *y*-axis, depicts the occurrence of overfitting in the model.

## 3. Results and Discussion

The proposed approach indulged RNN and LSTM classifiers to forecast the location of membrane proteins within the cell, and for this comparison, feature extraction was first performed through PseAAC. This appropriate information of amino acids as relevant features was input to the proposed models of LSTM and RNN, with factual dropout values in every model yielding an accuracy of 83.4% and 80.5%, respectively, as figured out in Table 3.

**Figure 6:**
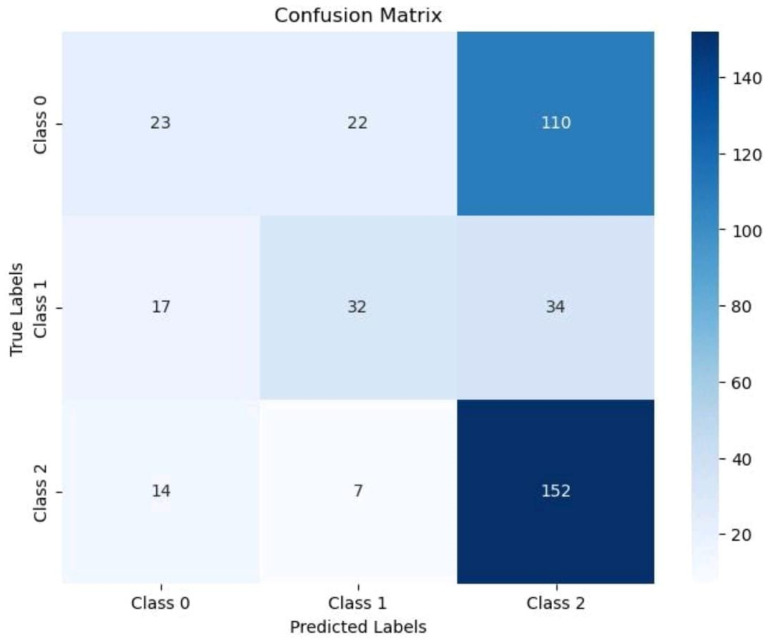
Confusion matrices for Long Short-Term Memory (LSTM) and Recurrent neural network (RNN) implemented on membrane proteins for tagging their location.

A confusion matrix is the key performance indicator of the implemented algorithms, depicting the values of all the parameters critical for precise outcomes. The 3 × 3 confusion matrices of Figure 6 reveal the working of the classifiers individually in categorizing membrane proteins into one of the three different locations.

The confusion matrix offers a tabular summary comparing the model’s predictions and actual results; however, it is not sufficient to depict the overall performance of the deep learning models. Therefore, certain other evaluators of the performance of deep learning models are extracted from the confusion matrix based on the values of true positive (TP), false positive (FP), false negative (FN), and true negative (TN) as mentioned in Table 4.

**Figure 7:**
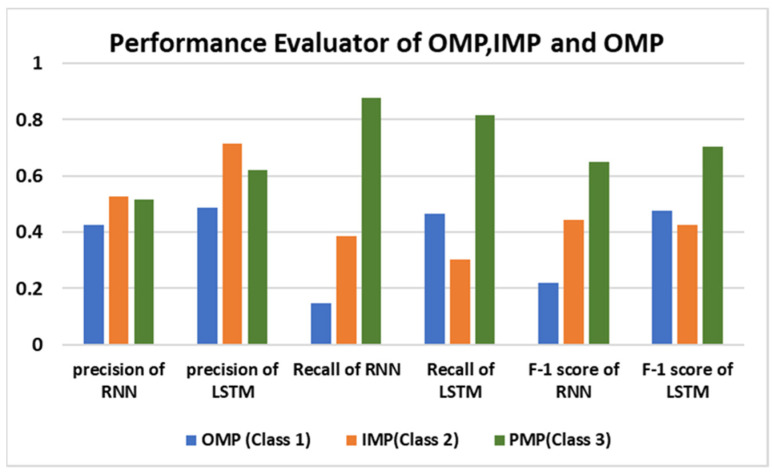
Performance evaluators for LSTM and RNN for membrane proteins location recognition where OMP = organelle membrane proteins, IMP = internal membrane proteins, and PMP = plasma membrane proteins.

The above Table 4. compares the various performance evaluators, including precision, negative predictive value (NPV), false positive rate (FPR), sensitivity, specificity, and accuracy for the deep learning models. The LSTM model demonstrates a higher precision of 71.4% compared to the RNN model, effectively reducing the incidence of false positives in predicting potential membrane proteins. Such errors are particularly concerning in drug development, where incorrect protein localization can lead to ineffective therapies or adverse effects. Additionally, LSTM achieves a specificity of 96.9%, indicating that a protein predicted to be non-membrane localized is highly unlikely to actually be membrane localized. In contrast, the RNN model exhibits a specificity of 91%, implying that it may misclassify some non-membrane proteins as membrane localized. A further deep dive into the confusion matrices of the RNN and LSTM discloses the values of recall, precision and F-1 score for three classes of OMP, IMP, and PMP for a better comparison, as depicted in Figure 7.

The above figure shows that the LSTM model performs much better than RNN in the classification of organelle membrane proteins (Class 1), most notably in recall. In particular, the LSTM yields a recall of 0.464, while the RNN has a recall of 0.148. The salient difference indicates that LSTMs are able to learn long-range dependencies within the sequence data more effectively, allowing for more consistent classification of Class 1 instances. On the other hand, for Class 2 of internal membrane proteins the LSTM outperforms the precision (0.714 vs. 0.525), meaning that when the LSTM predicts Class 2, its predictions are more likely to be correct. On the other hand, LSTM has a lower recall for Class 2 (0.301 vs. 0.386), indicating that the model misses more internal membrane proteins. However, it shows that although the LSTM does better than the RNN in predicting Class 2 positives correctly, it is still failing to capture all the relevant instances, which is a common problem for both methods. Both of the models perform well for Class 3 of plasma membrane proteins and the LSTM outperforms the RNN in terms of precision (0.619 vs. 0.514) and F1-score (0.705 vs. 0.648). However, RNN gains a slightly high recall (0.879 vs. 0.815), where it may be better at finding Class 3, but at a lower level of precision. In contrast, the LSTM achieves better performance across precision and F1-score. The output is probably due to the LSTM better modeling long-term dependencies in sequential data compared to the RNN. In conclusion, the LSTM model has an improvement in accuracy when compared to RNN. This indicated that the relatively better overall performance of LSTM is due to it being more able to capture long-range dependencies and able to handle complex sequence data.

For comparison of results attained from proposed DL models, there exists no pre-defined criteria, as no prior study has employed these two distinct deep learning models in the same study for the analysis of membrane proteins. Furthermore, there is a scarcity of membrane protein location identifiers derived from deep learning methodologies. [21,22]. SCLpred-MEM [21] adopted a deep N-to-1 convolutional neural network (N1-NN) to dig out the location of membrane proteins with an accuracy of 81.25%; however, the drawback is that it only reveals whether the protein belongs to the category of membrane proteins or not. The proposed CNN network in this study attained an accuracy of 82.4% by exposing the site of membrane proteins in three distinct places. Membrane proteins are recognized based on images obtained from cryo-electron tomography [22], thereby rendering comparisons with studies relying on amino acid sequence-based data as proposed in this DL-based investigation unfeasible. Another research by Yushuang et al., by incorporating MLFE classifiers with blending of multiple diverse features on the SARS-CoV-2 dataset, yielded an accuracy of 72.73% in revealing the locale of proteins, not the membrane proteins.

The outcomes of the proposed models are correlated with other approaches having the same MemLoci dataset. When the performances of trained models were compared, it became clear that the deep learning models performed significantly better than the previously applied machine learning technique SVM [10]. The contrasting values of accuracies for various location predictors with distinct machine learning algorithms for proteins are depicted in Table 5.

The analysis of the deep learning models’ outcomes for RNN and LSTM depicts that the accuracies are 80.5% and 83.4%, respectively. The difference in predictive abilities of these algorithms points out the better performance of LSTM in protein locale identification. Moreover, less accuracy might be associated with gradients in standard RNNs, which makes it hard for the network to learn the relevant information hidden in the long chain of amino acids.

## 4. Conclusions

Membrane proteins are the primary class of targets for medicines and have essential physiological functions in vivo. Investigation of membrane proteins has a significant impact on the development of drugs since most medications interact with the membrane protein to produce their therapeutic effect. Thus, the correct locale information of these proteins depicts their proper functionality, thereby aiding in the precise operation of newly discovered drugs. To expedite this medication design process for new treatments against the diseases ravaging the world, deep learning techniques are the best fit to deal with a gigantic number of proteins. Therefore, this comparative study is performed on unfolding the locale of membrane proteins into three specific points in the cell to investigate the performance of deep learning algorithms’ approaches on a standardized dataset from SwissProt. The amalgamation of pseudo amino acid composition in the auto-construction of the feature selection mode of DL takes account of hydrophobicity and hydrophilicity values through PseAAC, thus extracting better clues about the locale of MP. The performance of the models is assessed by confusion matrices investigating the pattern of accuracy and losses during the training phase of the models and the accuracy attained. The outcome shows that the LSTM model outperformed RNN in declaring the residing site of membrane proteins as the plasma membrane, interior membrane, or membrane of an organelle. This allows for the automatic classification of newly identified membrane proteins just through the input of their amino acid sequences, thus facilitating the classification of these membrane proteins. This approach shows promising results overall, but the indulgence of membrane topology and signal peptide can further improve the outcomes. In the future, these factors can be included with deep learning to understand membrane protein localization with better outcomes. Moreover, the location identification of membrane proteins has not been accomplished using transfer-based deep learning models, which is another research question that needs to be solved.

## Figures and Tables

**Table 1:** Review of deep learning algorithms implemented.

Authors	Database	Deep Learning Algorithm	Performance Accuracy
Almagro Armenteros et al. [15]	UniProt database	RNN	92% (membrane-protein)
Hou et al. [16]	SM424-18 dataset	CNN	Not reported
Savojardo et al. [17]	SM424-18 dataset	CNN	Not reported
Thumuluri et al. [18]	UniProt database release 2021_03, Human Protein Atlas	protein transformer language model	Not reported
Liao et al. [19]	D3106, D4802	BLSTM	Not reported
Kaleel et al. [21]	UniProtKB release 2019_05	Ensemble of Deep N-to-1 Convolution Neural Network	81.25%
Lamm et al. [22]	cryo-electron tomograms	Convolution Neural Network	Not reported
Pan et al. [23]	SwissProt	RNN	86.9
Shah et al. [24]	NCBI protein database	1D-CNN	In the range of 93.24–97.30%, for SIRT1 to SIRT7

**Table 2:** Hyperparameter settings for the proposed models.

	RNN	LSTM
DL Model Layers	3	3
No. of Dense Layers	2	3
No. of Dropout Layers	4	5
Loss Function	Cross-Entropy	Cross-Entropy
Training Loss	0.56	0.70
Validation Loss	0.60	0.66
Testing Loss	0.66	0.76
Training Accuracy	0.841	0.81
Validation Accuracy	0.852	0.800
Testing Accuracy	0.832	0.80

**Table 3:** Outcomes for the proposed inter-classifier comparison of the DL model.

Implemented Models	Accuracy
LSTM	83.4%
RNN	80.5%

**Table 4:** Interpretation of performance of the deep learning models.

	Precision (%)	NPV (%)	FPR (%)	Sensitivity (%)	Specificity (%)	Accuracy (%)
**LSTM**	71.4	84.5	3	30.12	96.9	83.4
**RNN**	52.4	85.4	8.8	38.5	91.1	80.5

**Table 5:** Comparison of outcomes of various predictors with our proposed model.

Methods	Accuracy (%)
MemLoci [10]	70
Proposed RNN Model	80.5
Proposed LSTM Model	83.4
SCLpred-MEM [21]	81.25
MemBrain [22]	82.4
MLFE Classifier [26]	72.73

## Data Availability

The benchmark dataset of membrane proteins is available at https://mu2py.biocomp.unibo.it/memloci/ (accessed on 5 January 2023).
